# Comparing the Time-Dependent Evolution of Microcirculation in Gracilis vs. ALT Flaps Using Laser-Doppler Flowmetry and Tissue-Spectrometry

**DOI:** 10.3390/jcm11092425

**Published:** 2022-04-26

**Authors:** Nicholas Moellhoff, Paul I. Heidekrueger, Konstantin Frank, Svenja Pistek, Verena Alt, Riccardo E. Giunta, Denis Ehrl

**Affiliations:** 1 Division of Hand, Plastic and Aesthetic Surgery, University Hospital, Ludwig Maximilian University of Munich, 81377 Munich, Germany; nicholas.moellhoff@med.uni-muenchen.de (N.M.); konstantin.frank@med.uni-muenchen.de (K.F.); svenja.pistek@campus.lmu.de (S.P.); verena.alt@med.uni-muenchen.de (V.A.); riccardo.giunta@med.uni-muenchen.de (R.E.G.); 2 Centre of Plastic, Aesthetic, Hand and Reconstructive Surgery, University of Regensburg, 93053 Regensburg, Germany; paul@heidekrueger.net

**Keywords:** microvascular flow, O2C, anastomosis, free flap, microsurgery

## Abstract

Postoperative free flap monitoring is considered a key component of care after microsurgical reconstruction. To achieve successful flap salvage after surgical revision, early recognition of vascular compromise is required. The aim of this study was to assess and compare the time-dependent evolution of microcirculation in gracilis muscle (GM) and anterolateral thigh (ALT) flaps. This study included continuous measurements of blood flow (flow), hemoglobin oxygenation (SO_2_) and the relative amount of hemoglobin (rHb) using laser-doppler flowmetry and tissue-spectrometry (O2C, LEA Medizintechnik, Gießen, Germany) over a time-period of 72 h. Microcirculation was assessed in a total of 66 viable free flaps (GM *n* = 40; ALT *n* = 26). A statistically significant positive correlation between time post-anastomosis and microvascular flow was found for both GM and ALT flaps with r_s_ = 0.384 (*p* < 0.001) and r_s_ = 0.178 (*p* = 0.015), respectively. No significant positive or negative correlations between time post-anastomosis and SO_2_ were found for both GM and ALT flaps with r_s_ = 0.052 (*p* = 0.387) and r_s_ = −0.018 (*p* = 0.805), respectively. Overall, a significant negative correlation between time post-anastomosis and rHb was found for GM flaps with r_s_ = −0.140 (*p* = 0.019). For ALT flaps, no significant positive or negative correlation was found with r_s_ = −0.011 (*p* = 0.887). Microcirculation differs in different flap entities, and surgeons should be aware of these differences in order to correctly evaluate and classify the values of flow, SO_2_ and rHb obtained when using the O2C device for postoperative monitoring.

## 1. Introduction

Postoperative free flap monitoring is considered a key component of postoperative care after microsurgical reconstruction performed by both plastic surgeons and surgical nurses. Early recognition of vascular compromise is essential, in order to achieve successful flap salvage after surgical revision [1]. In addition to clinical monitoring of color, capillary refill time, turgor and temperature, technical devices exist to reduce human error and to objectify the status of free flap perfusion by measuring microvascular flow (flow), hemoglobin oxygenation (SO_2_) or the relative amount of hemoglobin (rHb) in the capillary bed [2,3,4].

Previously, our study group investigated the microcirculation of viable free flaps using laser-doppler flowmetry and tissue-spectrometry [5]. Continuous measurements over a time period of 72 h post-anastomosis revealed distinct perfusion dynamics, which can be related to the physicochemical mechanisms such as vasodilatation, hyperemia and increased oxygen consumption encountered during ischemia and reperfusion [3,4,6]. It was shown that overall, mean values of flow increased significantly over time, while SO_2_ showed a decreasing trend line and rHB remained constant throughout the study period. However, a main limitation of the study was the heterogeneity of free flaps investigated, including fasciocutaneous, musculocutaneous and muscle flaps. Importantly, it was acknowledged that each flap entity might have an individual hemodynamic profile. This can be related to rheologic effects of different tissue composition, as well as differences in vascular supply patterns and variability in vessel calibers [7,8,9,10,11,12,13].

Exemplary, according to Mathes and Nahai, free muscle, musculocutaneous and fasciocutaneous flaps can be categorized into different groups depending on their vascular supply [14]. The gracilis muscle (GM) flap is considered a type II flap, as it is supplied by a dominant, and one (or more) minor pedicles [14]. The anterolateral thigh (ALT) flap is considered a type B or type C flap, with a septocutaneous or musculocutaneous perforator [15]. Following these different categorizations, there might also be differences in the parameters of microcirculation measured between different free flap entities. 

Hence, the aim of this study was to assess and compare the time-dependent evolution of microcirculation of two frequently utilized free flaps, namely GM and ALT flaps, using continuous measurements of laser-doppler flowmetry and tissue-spectrometry over a time-period of 72 h post-anastomosis.

## 2. Materials and Methods

### 2.1. Study Design

This study was designed as a prospective single-center study to compare the evolution of microcirculation in two different flap types, gracilis muscle (GM) flaps and anterolateral thigh (ALT) flaps. The study was conducted at a level 1 hospital in Germany (University Hospital, LMU Munich) between 2020 and 2022. All free flap surgeries were performed by the senior author (D.E.). Ethical approval was granted by the local institutional review board (IRB protocol number: 20-549). 

### 2.2. Sample

Upon availability of the O2C device (LEA Medizintechnik, Gießen, Germany), all patients requiring GM or ALT free flap reconstruction—irrespective of defect etiology and localization—treated at the Division of Hand, Plastic and Aesthetic Surgery of the University Hospital, LMU Munich were included in the study. Patients’ incapable of understanding the aims and scope of the study and/or under the age of 18 were excluded from the study. No further exclusion criteria were defined. As this study investigated microcirculation in viable flaps only, flaps with major complications (defined as total flap loss or partial flap loss of >10%), and flaps requiring emergent revision surgery (i.e., arterial or venous thrombosis or hematoma) were excluded from data analysis. In order to compare a muscle flap (GM) with a fasciocutaneous flap (ALT) only, ALT flaps incorporating vastus lateralis muscle (myocutaneous vastus lateralis flaps) were also excluded from analysis. 

### 2.3. Assessments and Outcomes

Microcirculation was continuously measured using the O2C device and the LFx37 probe (both LEA Medizintechnik, Gießen, Germany) according to a previously described protocol [5]. Briefly, the O2C device measures blood flow (flow), hemoglobin oxygenation (SO_2_) and the relative amount of hemoglobin (rHb) within the capillary-venous compartment of the vascular tree using a laser-doppler flowmetry and a tissue-spectrometry-unit [16]. For GM flaps, the probe was sutured directly on to the muscle, while for ALT flaps it was attached to the skin island using medical device proofed double-sided tape. The probe was placed as far distally from the vascular pedicle as possible. The time of microvascular anastomosis was noted, and measurements commenced immediately postoperatively for a period of 72 h post-anastomosis. Measurements were performed continuously and were only interrupted occasionally for patient transportation, probe dislocation, or to correct signal interferences due to blood or wound exudate collecting underneath the measuring probe. 

### 2.4. Data Extraction and Statistical Analysis

For each free flap, mean values of microvascular flow, SO_2_ and rHb were extracted in hourly intervals over a period of 72 h post-anastomosis, using the O2CevaTime Software (Version No. 28.3, LEA Medizintechnik, Gießen, Germany). The time-dependent course of the variables was analyzed based on the following reference times: 1, 3, 6, 12, 24, 36, 48, 60, and 72 h post-anastomosis. Data are presented as means with respective standard deviation (1 SD). Data were tested for normal distribution using the Shapiro–Wilk test and by visual inspection of normal Q-Q plots. Data were normally distributed, and differences between the two groups (GM vs. ALT) at the respective time intervals were assessed using the unpaired Student’s t-test. A Spearman’s rank-order correlation was run to assess the relationship between the time post-anastomosis at the respective reference times and the three parameters of microcirculation (flow, SO_2_, rHb). For all analyses, the level of statistical significance was set at *p* < 0.05 to guide conclusions. All statistical analysis was conducted in SPSS Statistics 28 (IBM, Armonk, NY, USA).

## 3. Results

Data of a total of 161 free flaps were extracted using the O2C device and appropriate software. This study then included continuous measurements of a total of 66 viable free flaps (GM *n* = 40; ALT *n* = 26) performed in 66 patients (43 male, 23 female) with a mean age of 60.94 ± 17.19 years (GM: 58.78 ± 17.34 years vs. ALT: 64.27 ± 16.74 years). 

### 3.1. Microvascular Flow

**Figure 1:**
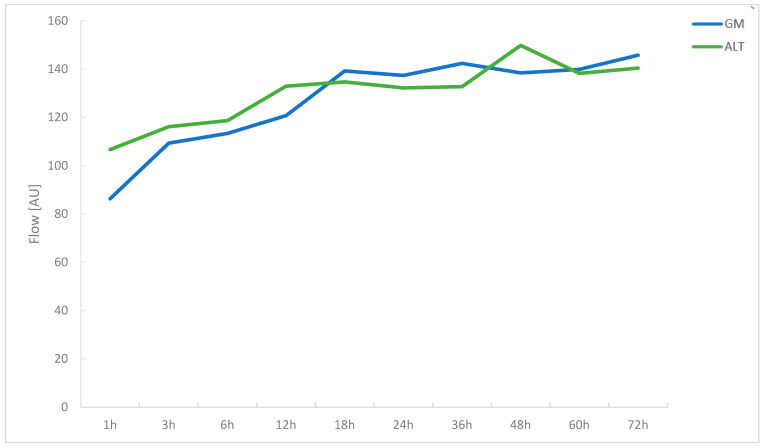
Line graph comparing microvascular blood flow (flow) in gracilis muscle and ALT free flaps over a period of 72 h post-anastomosis.

For both GM and ALT flaps, mean values of microvascular flow showed a strong increase over time after anastomosis (Figure 1). For GM flaps, values increased from 86.31 ± 24.98 A.U. to 145.77 ± 43.26 A.U., as measured from 1 to 72 h post-anastomosis. Within the same time period, flow increased from 106.67 ± 50.71 A.U. to 140.41 ± 48.64 A.U. in the ALT group. Peak measurements for flow were reached at 72 h post-anastomosis in the GM group (145.77 ± 43.26 A.U.), whereas they were reached after 48 h in the ALT group (149.84 ± 58.40 A.U.). Overall, microvascular flow in GM and ALT flaps evolved similarly over time, with no significant differences between mean values of flow at any of the investigated time intervals (Table 1). A statistically significant positive correlation between time post-anastomosis and microvascular flow was found for both GM and ALT flaps with r_s_ = 0.384 (*p*< 0.001) and r_s_ = 0.178 (*p* = 0.015), respectively.

### 3.2. Hemoglobin Oxygenation (SO_2_) 

**Figure 2:**
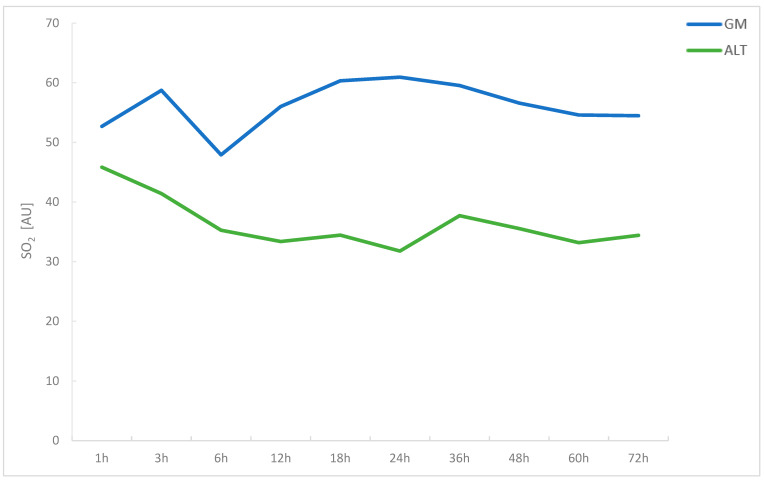
Line graph comparing hemoglobin oxygenation (SO_2_) in gracilis muscle and ALT free flaps over a period of 72 h post-anastomosis.

In the GM group, mean values of SO_2_ remained fairly constant over time. SO_2_ values evolved from 52.69 ± 25.70 A.U. at 1 h post-anastomosis to 54.46 ± 21.45 A.U. at 72 h post-anastomosis. A decreasing trend was found for SO_2_ values in the ALT group, as mean measurements at 1 h post-anastomosis were 45.83 ± 35.47 A.U. and decreased down to 34.41 ± 19.35 A.U. However, no significant positive or negative correlations between time post-anastomosis and SO_2_ were found for both GM and ALT flaps with r_s_ = 0.052 (*p* = 0.387) and r_s_ = −0.018 (*p* = 0.805), respectively. Overall, SO_2_ values in GM flaps were higher as compared to ALT flaps, with results differing significantly at 3, 12, 18, 24, 36, 48, 60, and 72 h post-anastomosis (all *p* < 0.05) (Table 2, Figure 2). 

### 3.3. Relative Amount of Hemoglobin (rHb) 

**Figure 3:**
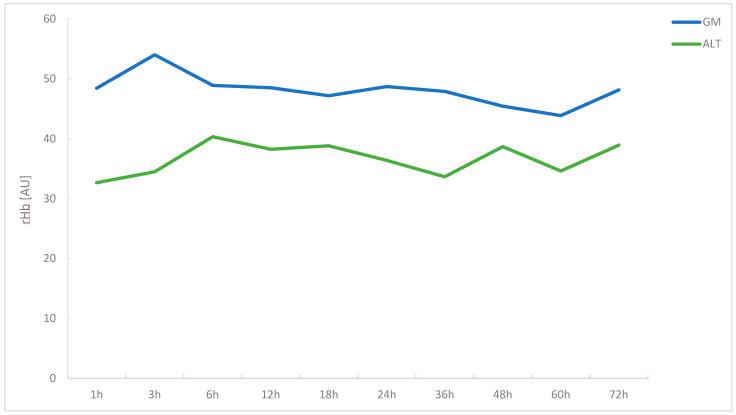
Line graph comparing relative amount of hemoglobin (rHb) in gracilis muscle and ALT free flaps over a period of 72 h post-anastomosis.

Values for rHb remained stable for GM flaps post-anastomosis. At 1 h post-anastomosis, values were as high as 48.44 ± 22.08 A.U., reaching 48.15 ± 16.30 A.U. at 72 h post-anastomosis. Values for ALT flaps increased from 32.67 ± 7.26 A.U. to 38.94 ± 20.98 A.U. over the investigated study period. Overall, a small but significant negative correlation between time post-anastomosis and rHb was found for GM flaps with r_s_ = −0.140 (*p* = 0.019). For ALT flaps, no significant positive or negative correlation was found with r_s_ = −0.011 (*p* = 0.887). Similar to the results presented for SO_2_, overall rHb values in GM flaps were higher as compared to ALT flaps, reaching significance at 3, 24, 36 and 60 h post-anastomosis (all *p* < 0.05) (Table 3, Figure 3).

## 4. Discussion

This study assessed and compared the physiological perfusion dynamics in a patient cohort receiving two different types of free flaps using laser-doppler flowmetry and tissue-spectrometry measurements provided by the O2C monitoring device (LEA Medizintechnik, Gießen, Germany). For the first time, the data show parallels and differences in microvascular flow, SO_2_ and rHb between GM and ALT flaps in the first 72 h post-anastomosis. The results of this study thus add further information regarding the time-dependent course of physiological microcirculation after re-anastomosis, and provide guidance to surgeons using the O2C device for postoperative flap monitoring as to what to expect in the early postoperative period. To summarize, the data show that microvascular flow developed comparably in GM and ALT flaps, both increasing over the study period with a statistically significant positive correlation between time post-anastomosis and microvascular flow. This is in line with pooled data of various flap types presented by our study group previously, which revealed that flow significantly increased up to 18 h post-anastomosis, after which peak formation occurred [5]. Interestingly, in GM and ALT flaps the peak measurements for flow were reached at 72 h post-anastomosis, whereas they were reached after 48 h in the ALT group. Increases of microvascular flow post-anastomosis have been attributed to ischemia and reperfusion, which induce hyperemia, vasodilatation, and a decrease of vascular resistance based on physical (sympathectomy) and chemical (accumulation of anaerobic metabolites, inflammatory proteins, reactive oxygen species) effects [11,17,18,19,20,21,22]. Free flap vascular territories (angiosomes) are three-dimensional tissue units supplied by a distinct source artery [23]. Adjacent vascular territories are connected via choke vessels. This is of significant relevance in free flap surgery, as the axial arterial supply of angiosomes located in proximity to the territory supplied by the main (perforator-) pedicle might be cut or ligated during flap harvesting. Adequate tissue perfusion is then dependent on blood inflow via connecting choke vessels [24,25]. The impact of choke vessel dilation on the parameters of microcirculation remains to be established. Studies have determined that choke vessel dilation is not an immediate consequence of perforator ligation, but occurs between 24 and 72 h after flap elevation [24,26]. Dilation is connected to arterial inflow, with increased blood flow supporting choke vessel dilation [24]. The increase of microvascular flow observed in both flap entities in this study over 72 h might therefore enable choke vessel dilation, thereby decreasing vascular resistance and promoting free flap viability. 

Differences between the two investigated groups were found with regard to SO_2_ and rHb values, as both were significantly higher in GM flaps during the investigated time periods. Over time, a decreasing trend was found for SO_2_ values, together with an increasing trend for rHb values in the ALT group, without, however, showing a statistically significant correlation over time. In the GM group, overall values for SO_2_ and rHb remained constant, with a small but significant negative correlation between time post-anastomosis and rHb. Hölzle et al. investigated microcirculation in radial forearm flaps, which—similar to the ALT flap—is a fasciocutaneous flap [3]. Different from our approach, they performed interrupted measurements of flow, SO_2_ and rHb at 1, 3, 7 and 14 days postoperatively. In line with the presented data, they found an increase of flow post-anastomosis, attributed to a hyperemic response to tissue hypoxia. In addition, they described stable values for SO_2_ after anastomosis, which decreased by the third postoperative day, while hemoglobin concentration remained stable [3]. Similarly, while Spearman´s correlation showed no significant negative trend between time post-anastomosis and SO_2_, the absolute values of SO_2_ in the ALT group decreased from 45.83 ± 35.47 A.U. to 34.41 ± 19.35 A.U in our study population. Contrary, absolute values of rHb increased from 32.67 ± 7.26 A.U. to 38.94 ± 20.98 A.U. over the 72 h follow-up in ALT flaps, although once more Spearman´s Rho showed no significant correlation. In a follow-up study, Hölzle et al. compared radial forearm flaps with fibular, perforator and ALT flaps, and found significantly higher SO_2_ and flow values in forearm flaps [4]. They attributed this to the fact that the radial forearm is supplied by many closely meshed fasciocutaneous vessels, while the lateral leg is supplied by single septocutaneous or myocutaneous vessels [4].

From our experience, probe placement has a strong impact on the measurement parameters. Placing the probe at a different location, and with altered pressure, can largely affect values for flow, SO_2_ and rHb. It is hard to believe that in the aforementioned studies the probe was placed at the exact same location at every measuring time point of the interrupted measurements. Therefore, the values might have differed between the investigated time points solely due to inconsistent probe placement. Hence, we consider the standardized and continuous probe placement that we performed as a significant strength of our study.

There is a scarcity of studies comparing fasciocutaneous with muscle flaps with regard to the time-dependent evolution of the parameters relevant for microcirculation with the O2C device. Rahmanian-Schwarz et al. demonstrated superior thermoregulation in LDM flaps compared to ALT flaps in the postoperative course when exposed to hot and cold water, as assessed by measuring microvascular flow and velocity using the O2C device [27]. While not entirely relevant to our study, the data underline differences in postoperative microcirculation with regard to the type of free flap transplanted and show significant differences depending on the type of tissue incorporated in the flap. The authors speculate that the presence of the muscle in the LDM flap improves neural and vascular regeneration, thus offering better conditions for thermoregulation [27]. For both flaps, measurements were however performed on the skin island, as the LDM flap was harvested as a myocutaneous flap. Therefore, it remains unanswered what measurements directly on the muscle, such as those performed in GM flaps in our study, would have revealed.

The results presented in our study demonstrate higher SO_2_ and rHb values in GM flaps, compared to ALT flaps, while no significant differences were found for microvascular flow. In our opinion, this could be the result of higher oxygen consumption in ALT flaps or greater capillary oxygen supply in GM flaps. Potentially, tissue hypoxia is more pronounced in fasciocutaneous compared to muscle flaps. This could result in higher post-anastomosis tissue oxygen consumption in ALT flaps, as compared to GM flaps, potentially explaining the differences in SO_2_. However, it is known that muscle tissue is less resistant to ischemia, compared to skin and fascia [28,29,30]. Therefore, it may be speculated that due to unspecified local regulatory mechanisms, intracapillary hemoglobin oxygenation could be increased in GM flaps. Thus, further studies investigating the ischemia tolerance of different flap entities are warranted and could potentially provide an explanation for the findings of this study.

From a clinical perspective, we observe higher levels of postoperative edema in patients receiving GM flaps, compared to ALT flaps. This is in line with literature, where postoperative swelling in muscle flaps is frequently described, resolving only after several months postoperatively in many cases [31,32,33]. This, in turn, could explain the higher levels of rHb found in GM flaps, as outflow in the capillary tree could be affected by the increased swelling. Ischemia-reperfusion injury causes leukocyte infiltration, inflammation, and an increase in interstitial edema and apoptosis [22,34]. Thus, postoperative swelling in muscular tissue is likely to be elevated, as it is more prone to ischemia in the first place [28,29,30]. Future studies should investigate the level of postoperative edema using standardized measuring devices such as three-dimensional imaging, to further elucidate the impact of postoperative edema on the parameters of microcirculation in different flap types.

This study marks the beginning of our effort in defining hemodynamic profiles in different flap entities. Next, given sufficient case numbers, investigation of further flap entities will follow. In addition, the question remains whether an understanding of physiological perfusion dynamics in different free flaps using the O2C device can ultimately change clinical outcomes and optimize clinical decision making with regard to emergent revision surgery upon vascular compromise. This remains to be elucidated in due course and can be considered a major shortcoming of this study. In addition, the impact of individual patient characteristics and co-morbidities on the parameters of microcirculation still remains elusive and needs to be addressed in the future. A further limitation is the lack of long-term data acquisition. It would be interesting to investigate the evolution of microcirculation in different flap entities also over an extended time-period, i.e., 3, 6 and 12 months postoperatively, to evaluate the impact of neovascularization and neoangiogenesis, making blood supply independent of the vascular pedicle. Lastly, the method of probe fixation (suture vs. tape) might influence the measurements obtained. However, we do believe that firm contact of the probe with the tissue is essential for reliable read-outs and the impact of the type of fixation is likely to be negligible if firm contact is achieved.

## 5. Conclusions

This study provides data to further define the hemodynamic profile and time-dependent perfusion dynamics in GM and ALT flaps post-anastomosis. Microcirculation differs in different flap entities and surgeons should be aware of these differences, in order to correctly evaluate and classify the values of flow, SO_2_ and rHb obtained when using the O2C device for postoperative monitoring. 

## Figures and Tables

**Table 1:** Detailed analysis of the evolution of microvascular blood flow (flow) in gracilis muscle and ALT free flaps over a period of 72 h post-anastomosis.

Time Post-Anastomosis (Hours)	Gracilis Muscle		ALT		*p*-Value
	Mean Flow (A.U.)	Standard Deviation	Mean Flow (A.U.)	Standard Deviation	
1	86.31	24.98	106.67	50.71	0.217
3	109.3	41.93	116.12	39.92	0.583
6	113.36	38.85	118.65	45.86	0.644
12	120.74	40.38	132.92	44.70	0.275
18	139.24	41.02	134.70	36.98	0.672
24	137.34	39.39	132.22	41.60	0.667
36	142.35	36.74	132.75	38.33	0.365
48	138.41	32.11	149.84	58.40	0.398
60	139.88	38.06	138.22	50.70	0.904
72	145.77	43.26	140.41	48.64	0.756

**Table 2:** Detailed analysis of the evolution of hemoglobin oxygenation (SO_2_) in gracilis muscle and ALT free flaps over a period of 72 h post-anastomosis.

Time Post-Anastomosis (hours)	Gracilis Muscle		ALT		*p*-Value
	Mean SO_2_ (A.U.)	Standard Deviation	Mean SO_2_ (A.U.)	Standard Deviation	
1	52.69	25.70	45.83	35.47	0.62
3	58.73	16.62	41.41	27.56	0.008
6	47.91	21.34	35.26	27.03	0.056
12	56.00	17.05	33.36	24.52	<0.001
18	60.32	18.86	34.43	24.71	<0.001
24	60.94	17.95	31.78	18.84	<0.001
36	59.53	17.14	37.70	21.41	<0.001
48	56.59	17.39	35.53	20.10	<0.001
60	54.58	17.58	33.17	23.15	0.001
72	54.46	21.45	34.41	19.35	0.012

**Table 3:** Detailed analysis of the evolution of relative amount of hemoglobin (rHb) in gracilis muscle and ALT free flaps over a period of 72 h post-anastomosis.

Time Post-Anastomosis (Hours)	Gracilis Muscle		ALT		*p*-Value
	Mean rHb (A.U.)	Standard Deviation	Mean rHb (A.U.)	Standard Deviation	
1	48.44	22.08	32.67	7.26	0.106
3	54.03	17.03	34.47	15.19	<0.001
6	48.91	16.47	40.35	23.70	0.116
12	48.51	15.99	38.24	24.03	0.051
18	47.18	14.93	38.83	24.10	0.112
24	48.72	13.69	36.39	14.02	0.004
36	47.91	13.22	33.65	15.21	0.001
48	45.44	13.80	38.68	14.82	0.12
60	43.88	13.31	34.61	15.91	0.047
72	48.15	16.30	38.94	20.98	0.201

## Data Availability

Restrictions apply to the availability of these data.
